# Effectiveness of the nucleus SmartNav system in detecting and preventing electrode tip fold-overs: a multi-centre retrospective analysis of 213 cochlear implantations

**DOI:** 10.1017/S0022215125104155

**Published:** 2026-05

**Authors:** Mehmet Ekrem Zorlu, Emirhan Akyol, Secaattin Gülşen, Recep Karamert, Mehmet Birol Ugur, Hakan Tutar

**Affiliations:** 1 Department of Otolaryngology and Head & Neck Surgery, Gazi University Faculty of Medicine, Ankara, Türkiye; 2 Department of Otolaryngology and Head & Neck Surgery, Gaziantep City Hospital, Gaziantep, Türkiye

**Keywords:** cochlear implantation, SmartNav system, tip fold-over, X-ray, hearing loss

## Abstract

**Objectives:**

This study aimed to evaluate the effectiveness of the SmartNav in detecting tip fold-over during cochlear implantation and to compare angular insertion depth measurements obtained via SmartNav and transorbital X-ray imaging.

**Methods:**

This retrospective multicentre study included patients with normal cochlear anatomy, comprising 163 individuals and 213 ears who underwent cochlear implantation using Nucleus CI522 and CI622 systems at Gazi University Faculty of Medicine and Gaziantep City Hospital.

**Results:**

Of the 213 cochlear implantations, tip fold-over was detected in 4 implantations (1.88 per cent) intra-operatively with SmartNav. One case (0.47 per cent) of tip fold-over was not detected by SmartNav and identified post-operatively through X-ray imaging. SmartNav showed a sensitivity of 80 per cent, specificity of 100 per cent. A strong correlation was found between SmartNav and X-ray angular insertion depth measurements (*p* < 0.001).

**Conclusion:**

The SmartNav is a reliable tool for the intra-operative detection of tip fold-overand the assessment of angular insertion depth in patients with normal cochlear anatomy.

## Introduction

Cochlear implants have revolutionised the treatment of patients who have severe to profound sensorineural hearing loss and do not benefit from hearing aids. Several factors affect the success of implantation such as integrity of device, device function assessment and proper placement of the internal electrode array.^1^^,^^2^ The placement of the electrode array into the scala tympani is associated with superior audiological outcomes.^3^ In particular, the flexible perimodiolar arrays are more susceptible to tip fold-overs (TFOs).^2^^,^^4^ TFO especially occurs in the most apical electrodes and affects implant function by impeding the contact between the distal electrodes and apical cochlea.^2^ TFOs can be detected intra-operatively with imaging techniques such as X-ray imaging, fluoroscopy and computed tomography (CT). However, these modalities prolong the operation duration, increase the cost and expose the patient to radiation, while the additional safety measures, such as requiring the surgical team to temporarily leave the theatre, add logistical challenges and stress. X-ray imaging can also be used post-operatively to minimise anaesthesia time.

There are also electrophysiological measurements including stapedial reflex testing, electrical impedance and electrically evoked compound action potentials to ensure the optimal function and the appropriate placement of electrode arrays.^5^ Although these tests can accurately evaluate the function of the implant, they do not assure the proper placement of arrays.^2^

Nucleus SmartNav System is a tablet-based new measurement method released by Cochlear (Cochlear Ltd.,Sydney, NSW, Australia) in 2020. SmartNav analyses insertion speed, the angular insertion depth and ensures the proper electrode placement within the cochlea.^6^ This system utilises electrode voltage telemetry to assess the placement of the electrode array intra-operatively.^2^

This study aimed to analyse the utility of SmartNav in detecting TFOs intra-operatively in patients who have normal cochlear anatomy and compare the angular insertion depth measurements between SmartNav and X-ray.

## Materials and methods

Patients who underwent cochlear implantation using the SmartNav system between February 2024 and January 2025 at Gazi University Faculty of Medicine and Gaziantep City Hospital were included in the study. This retrospective multicentre study was approved by the Non-Interventional Clinical Research Ethics Committee of Gaziantep City Hospital. The study was conducted in accordance with the principles of the Declaration of Helsinki. Informed consent was obtained from all adult patients and the parents of paediatric patients.

**Figure 1.**
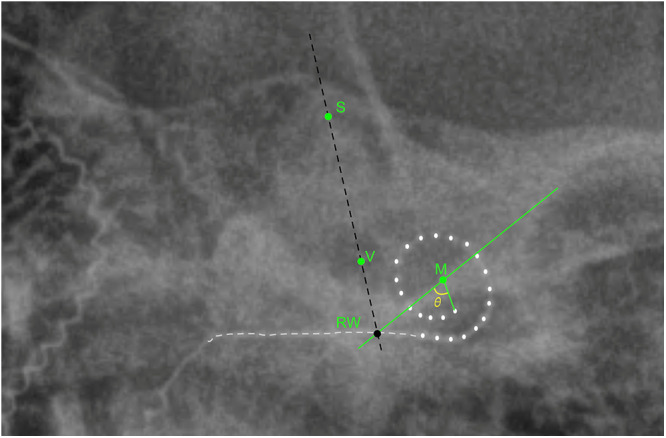
The method used to measure angular insertion depth. M, modiolus; RW, round window; S, superior semicircular canal; V, the center of the vestibule. A green line drawn between the modiolus and the round window represents the 0° reference line. Angular insertion depth is defined as the angle formed by the most distal electrode relative to this 0° reference line. Figure 1 long description.

In all patients, cochlear implantation was performed via a standard posterior tympanotomy/facial recess approach, with round window insertion. SmartNav was used for the angular insertion depth and insertion time measurements during round window insertion. After complete insertion of the electrode, a placement check was performed using SmartNav to evaluate the presence of TFO. Subsequently, impedance and AutoNRT measurements were carried out and recorded. TFO identified by SmartNav was confirmed intra-operatively using X-ray imaging. Post-operative transorbital X-ray imaging was performed to verify electrode position in all patients. Electrode positions were evaluated on X-ray images, and angular insertion depths were determined to assess the sensitivity of SmartNav in determining electrode position and measuring angular depth. Figure 1 illustrates the method used to measure angular insertion depth.

Angular insertion depth measurement^7^^,^^8^: Firstly, on the direct X-ray image, the apex of the superior semicircular canal (S) and the centre of the vestibule (V) are identified. A vertical line (dashed black line) is drawn between these two points; the intersection of this line with the electrode array approximately indicates the location of the round window (RW). Secondly, the modiolus (M) is identified as the centre of the spiral structure surrounded by the electrodes (white dots). A green line drawn between the modiolus and the round window represents the 0° reference line. Thirdly, angular insertion depth is defined as the angle formed by the most distal electrode relative to this 0° reference line. Fourthly, to evaluate inter-rater agreement, angular insertion depth measurements were independently repeated by a second researcher in a randomly selected subset of 30 patients. This sample size is consistent with methodological recommendations for assessing measurement reliability.^9^

Date of surgery, side of surgery, type of the surgery and the demographic data were collected. Insertion speed, the angular insertion depth, impedance and AutoNRT were recorded intra-operatively using the SmartNav system. The data obtained in the study were evaluated using IBM SPSS Statistics for Windows, Version 22.0 (IBM Corp., Armonk, NY, USA). The numerical variables were presented as mean plus or minus standard deviation (SD), while categorical variables were presented as numbers and percentages. Pearson correlation analysis was performed to evaluate the correlation between angular insertion depth measurements obtained from transorbital X-ray imaging and those provided by the SmartNav system. Sensitivity, specificity, positive predictive value and negative predictive value were calculated as defined by Tejani *et al*.^10^ Inter-rater reliability of angular insertion depth measurements was assessed using the intraclass correlation coefficient (ICC) with a two-way mixed effects model and consistency definition.

## Results

The present study consisted of 163 patients and 213 ears who underwent cochlear implantation between February 2024 and January 2025 at Gazi University Faculty of Medicine and Gaziantep City Hospital. Between these dates, a total of 269 ears in 208 patients received Nucleus cochlear implants. Of these, 17 were excluded due to inadequate post-operative transorbital X-ray images, 11 due to incomplete SmartNav measurements, 15 due to cochlear malformations and 2 due to cochlear ossification. Among these implantations included in the study, the CI522 model was used in 205 ears, while the CI622 model was used in 8 ears (Table 1). The mean age of the 163 patients included in the study was 17.12 plus or minus 20.90 years (min: 1; max: 69 years). Analysis of gender distribution revealed that 68 participants (41.7 per cent) were male, while 95 (58.3 per cent) were female. Of the 213 implants performed, 112 (52.6 per cent) were inserted on the right side and 101 on the left. The mean insertion time was 52.96 ± 27.05 seconds. Due to the lack of specific data from the SmartNav, the mean insertion time for cases in which TFO was detected could not be evaluated. The mean placement check time for SmartNav was 148.8 plus or minus 15.6 seconds. The mean angular insertion depth measured using SmartNav was 356.84° plus or minus 54.20°, while the mean angular insertion depth calculated from X-ray images was 377.47° plus or minus 41.19°. Correlation analysis between the two measurement methods revealed a strong positive correlation (r = 0.711; *p* < 0.001).

**Table 1.** Characteristics of the patients in the study Table 1 long description.

Characteristic	Value
No. of participants, n	163
No. of ears, n	213
Age (year),	
Mean ± SD	17.12 ± 20.9
Min–max	1−69
Gender, n (%)	
Female	95 (58.3)
Male	68 (41.7)
Cochlear implantation, n (%)	
Total	213 (100)
Right	112 (52.6)
Left	101 (47.4)
Cochlear implant model, n (%)	
CI522	205 (96.2)
CI622	8 (3.8)
Tip fold-over (TFO), n (%)	
SmartNav (-), X-ray (-)	208 (97.6)
SmartNav (-), X-ray (+)	1 (0.5)
SmartNav (+), X-ray (+)	4 (1.9)
SmartNav (+), X-ray (-)	-
SmartNav diagnostic accuracy, %	
Sensitivity	80
Specificity	100
Positive predictive value (PPV)	100
Negative predictive value (NPV)	99,5
Placement check time (seconds), mean ± SD	148,8 ± 15.6
Insertion time (seconds), mean ± SD	52.96 ± 27.05
Angular insertion depth (degrees), mean ± SD	
SmartNav	356.84° ± 54.20
X-Ray	377.47° ± 41.19

**Figure 2.**
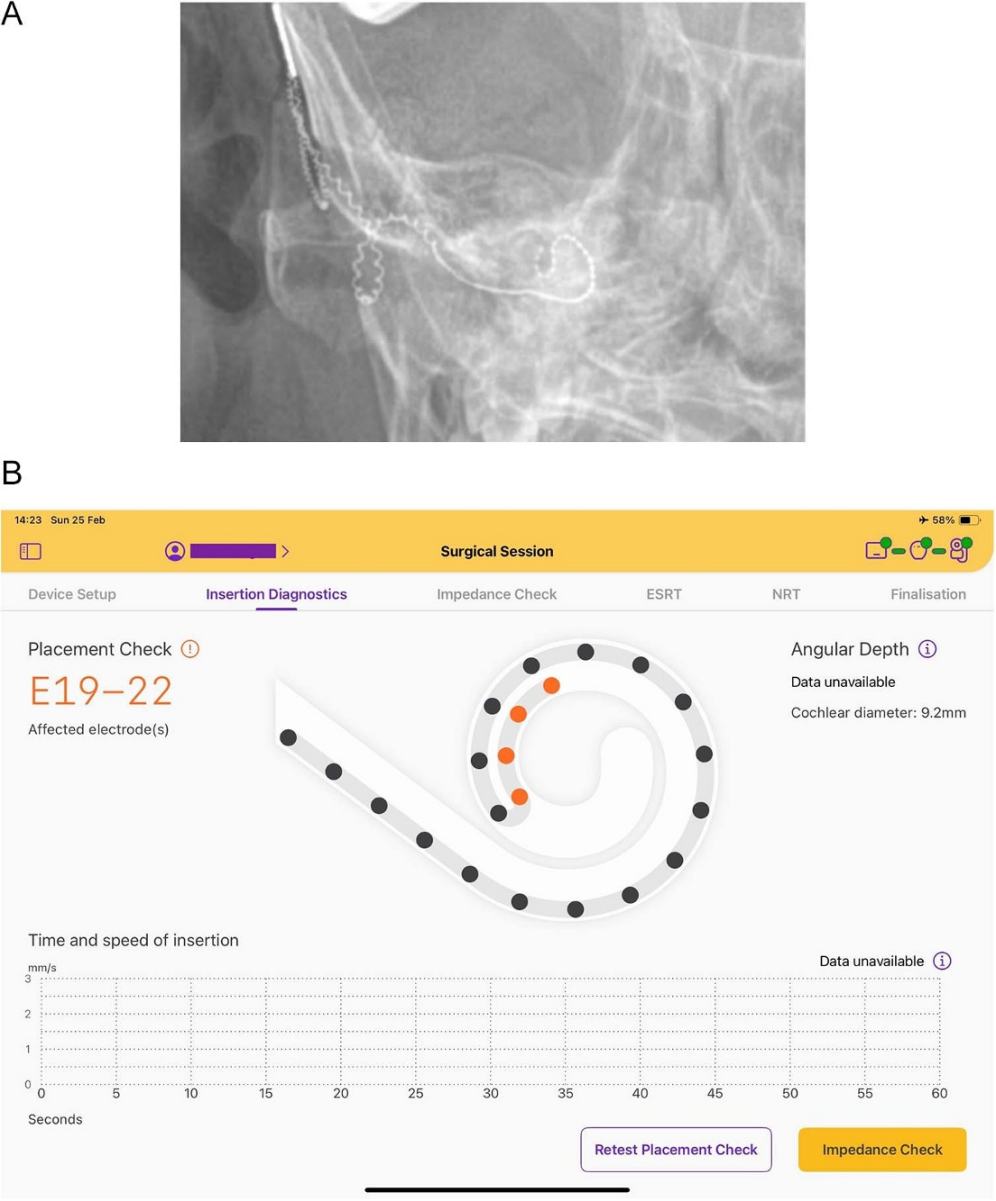
(A) Intra-operative X-ray image of a case in which TFO was identified. (B) SmartNav image of the same case. Figure 2 long description.

**Figure 3.**
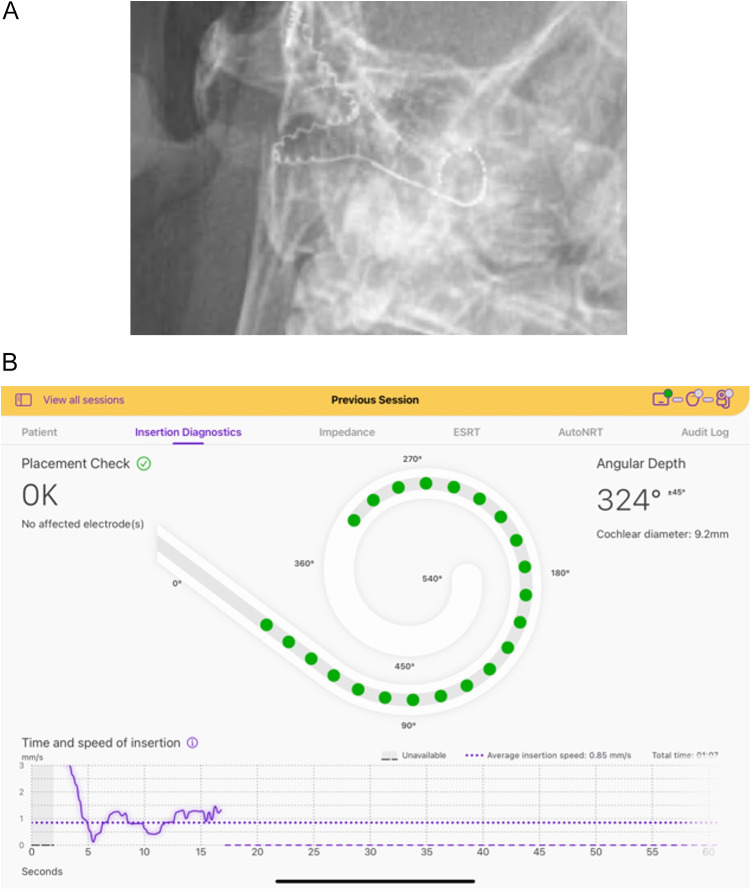
(A) The X-ray image following re-insertion of the electrode array in the aforementioned case. (B) SmartNav image after re-insertion of the electrode array. Figure 3 long description.

**Figure 4.**
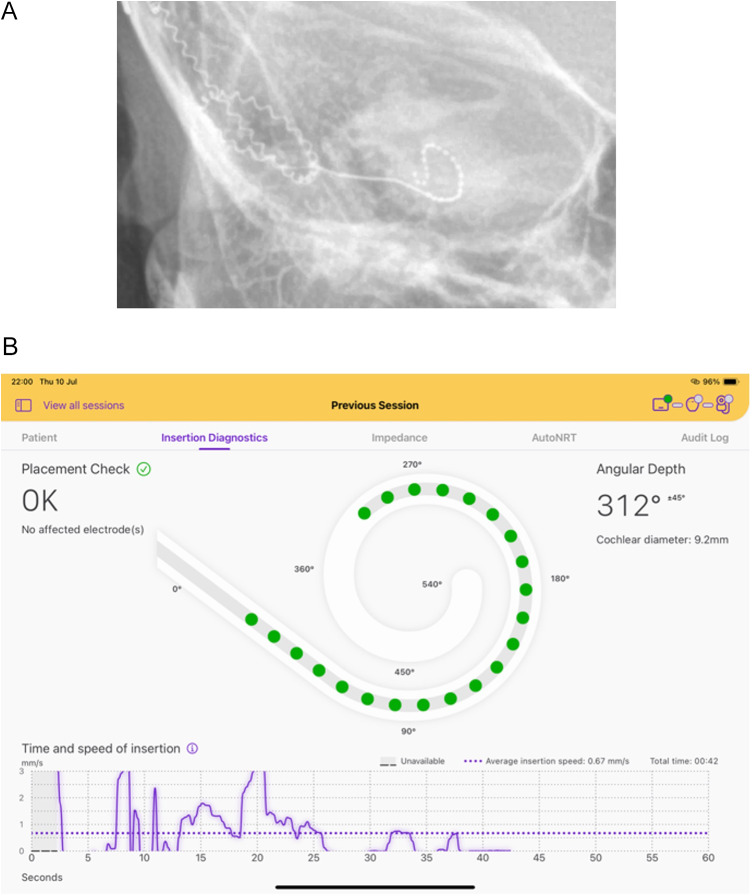
(A) Post-operative X-ray image of TFO which was not detected by SmartNav. (B) SmartNav image of the same case. Figure 4 long description.

Of 213 cochlear implantations, TFO was identified intra-operatively in 4 cases (1.88 per cent) during the placement check using SmartNav (Table 1). In all four cases in which TFO was detected using SmartNav, the findings were confirmed with X-ray imaging intra-operatively. The electrode arrays were pulled back and reinserted, resulting in proper positioning confirmed by both SmartNav and X-ray imaging. Figure 2A and 2B show intra-operative X-ray and SmartNav images of a case in which TFO was identified. X-ray and SmartNav images following re-insertion of the electrode array in the same patient are presented in Figure 3A and 3B. Post-operative X-ray imaging revealed TFO in one case (0.47 per cent), which was not detected by SmartNav (Figure 4A and B). When X-ray imaging is considered the gold standard to detect TFO,^11^ the SmartNav system demonstrates a positive predictive value of 100 per cent, a negative predictive value of 99.5 per cent, a sensitivity of 80 per cent and a specificity of 100 per cent.

Inter-rater reliability of the angular insertion depth measurements was evaluated in a randomly selected subset of 30 patients. The mean angular insertion depth measured by Rater 1 was 427.2° plus or minus 56.1°, while that measured by Rater 2 was 413.9° plus or minus 50.6°. To assess absolute agreement between raters, the ICC was calculated using a two-way mixed effects model with absolute agreement definition. The analysis yielded a single measure ICC of 0.942 (95 per cent CI: 0.573–0.985; *p* < 0.001), indicating excellent agreement between raters. The average measures ICC was 0.970, further demonstrating the high reliability of the average values obtained by the two raters.

## Discussion

In our retrospective study, the SmartNav system was found to effectively detect TFO in patients with normal cochlear anatomy, with a sensitivity of 80 per cent and a specificity of 100 per cent. The SmartNav system detected TFO intra-operatively in 4 cases (1.88 per cent) among 203 implantations during the placement check. In one case (0.47 per cent), SmartNav failed to detect TFO that was subsequently identified through X-ray imaging. Additionally, a strong correlation was observed between angular insertion depth measurements obtained from transorbital X-ray images and those provided by the SmartNav system.

Zuniga *et al.*^12^ identified TFO in 6 out of 303 implantations (1.98 per cent) using post-operative CT scanning. They also noted that all patients in whom TFO was detected had normal intra-operative telemetry measurements. Kay-Rivest *et al*.^11^ reported TFO rate of 2.5 per cent in their series of 117 cochlear implantations. Similarly, in our study, the TFO rate was 2.35 per cent, which is consistent with previously reported data. Lassig *et al*.,^13^ in their study on cochlear implant failures, reported that, in 13 per cent of reimplantation cases, poor performance was associated with suboptimal positioning of the electrode array. A misplaced electrode can compromise both hearing benefit and speech development and may also lead to complications such as vertigo and facial twitching due to stimulation of non-targeted regions within the cochlea.^5^^,^^14^

Techniques such as X-ray, fluoroscopy and CT can be utilised to detect TFO. Of the available options, X-ray imaging is the most commonly preferred due to its shorter procedure time, lower radiation exposure and greater accessibility compared to CT. Some authors recommend the routine intra-operative use of X-ray imaging or fluoroscopy.^11^^,^^15^ In addition, there are centres that evaluate the position of the electrode array intra-operatively through temporal CT.^16^ A major benefit of SmartNav is that it minimises the reliance on X-ray imaging. When used intra-operatively, X-ray imaging prolongs both the duration of surgery and anaesthesia. Intra-operative X-ray imaging and fluoroscopy can extend the duration of surgery by an average of 10 to 20 minutes.^2^^,^^15^ Prolonged anaesthesia duration may increase the risk of peri-operative complications, as well as long-term outcomes such as neurodevelopmental deficits.^2^^,^^17^^–^^19^ On the other hand, if X-ray imaging is performed after surgery and TFO is detected, a revision surgery is almost always required. Revision surgery leads to further exposure to anaesthesia, increased surgical risk, higher healthcare cost and the potential damage to the original implant, as well as considerable psychological stress for the surgical team, the patients and their families. In this context, the main advantage of SmartNav is its potential to reduce the number of missed intra-operative TFOs, rather than replacing imaging altogether. Another concern associated with X-ray imaging is radiation exposure. Repeated imaging may be necessary, which increases the patient’s cumulative radiation exposure and adds logistical burden and stress for the surgical team, as radiation safety procedures must be implemented multiple times. Interpretation of the imaging also requires experience.

In their cohort of 50 cochlear implantations, Cooper *et al*.^2^ detected TFO in 3 cases and concluded that SmartNav demonstrated a sensitivity of 100 per cent. They also reported that the mean time for SmartNav placement check was 2.12 minutes, compared to 14.23 minutes for intra-operative X-ray, indicating that SmartNav allows for a significantly faster verification of electrode position. In addition, they suggested that SmartNav enables faster and more straightforward interpretation of TFO than compared to X-ray imaging. Kelsall *et al.*^20^ also identified TFO in two patients within their series and reported that SmartNav demonstrated a sensitivity of 100 per cent in identifying TFO. Similarly, in their case series of 15 cochlear implantations, Kirbac *et al*.^5^ reported that the only TFO was successfully detected by SmartNav, noting a sensitivity of 100 per cent for the SmartNav system. Consistently, in a series of 113 cochlear implantations, Tejani *et al*.^10^ detected TFO in 6 patients, all of which were identified by SmartNav Placement Check, with both sensitivity and specificity reported as 100 per cent. Dangol *et al*.,^6^ in their study involving the use of the SmartNav system, reported that it accurately identified electrode position in all patients, reduced the duration of anaesthesia and facilitated determination of the optimal insertion speed required for slow and atraumatic electrode insertion. In contrast to previous studies, our findings showed that SmartNav failed to detect TFO in one case, with an overall sensitivity of 80 per cent and a specificity of 100 per cent.

In our routine practice, intra-operative X-ray imaging is reserved for patients with cochlear anomalies and is not routinely performed in those with normal cochlear anatomy. In patients without cochlear anomalies, X-ray imaging is performed post-operatively. Cooper *et al.*^2^ demonstrated that SmartNav can reliably detect TFO in patients with cochlear anomalies. More recently, Radomska *et al*.^21^ evaluated transimpedance matrix (TIM) measurements in children with various inner ear malformations and showed that characteristic TIM heatmaps can still be used to identify TFO even in these abnormal cochleae. However, current evidence remains limited, highlighting the need for further studies to assess the efficacy of SmartNav in patients with cochlear malformations.

In the present study, the mean duration of insertion was 52.96 plus or minus 27.05 seconds. Another advantage of the SmartNav system is its ability to provide real-time graphical feedback on the speed of electrode insertion. Previous studies have demonstrated that insertions exceeding 30 seconds are associated with improved hearing preservation outcomes.^22^ Faster insertions may cause mechanical trauma to the basilar membrane, potentially leading to scalar translocation and increasing the risk of TFO.^5^^,^^6^^,^^23^^,^^24^ Although rapid insertion time is believed to be associated with TFO, no analysis could be performed as insertion times could not be retrospectively retrieved for the cases in which TFO was detected. However, since the surgeons remained constant throughout the study, the mean insertion time in TFO cases is presumed to be similar.

Another advantage of the SmartNav system is that it eliminates the need for an audiologist during surgery, thereby contributing to workforce efficiency. This also allows audiologists to dedicate more time to follow-up patients, device programming and rehabilitation.^1^ Zawawi et al.,^1^ in a prospective study of 40 paediatric cochlear implantations utilising SmartNav, highlighted its contribution to increased surgical efficiency, shortened the duration of operation and its potential impact on auditory rehabilitation. However, in patients with abnormal cochlear anatomy, the involvement of an audiologist may still be required to overcome limitations of automated intra-operative systems.^1^^,^^25^

To the best of our knowledge, the present study includes the largest series involving the SmartNav system to date and presents the first comparison of angular insertion depth measurements between SmartNav and X-ray imaging.

The first limitation of our study is that it included only patients with normal cochlear anatomy, excluding those with cochlear anomalies. Further research is needed to evaluate the utility of SmartNav in such cases, following recent studies of TIM in malformed cochleae. Secondly, using CT instead of X-ray could have yielded more precise angular insertion depth measurements. Third, the vast majority of the implantations were performed with a lateral wall electrode (CI522) which is less prone to TFO, and the results may differ in other types of electrodes. The TFO rates in soft peri-modiolar electrodes like CI632 should be expected to be higher. In addition, SmartNav is currently applicable only to devices from Cochlear Corporation and not to implants from other manufacturers, which limits the generalisability of these workflow-related advantages.

- Nucleus SmartNav System is a tablet-based new measurement method released by the Cochlear Corporation. SmartNav analyzes insertion speed, the angular insertion depth and ensures the proper electrode placement within the cochlea.
- This study aimed to evaluate the effectiveness of the SmartNav in detecting tip fold-over (TFO) during cochlear implantation and to compare angular insertion depth measurements obtained via SmartNav and transorbital X-ray imaging.
- SmartNav showed a sensitivity of 80 per cent, specificity of 100 per cent, a positive predictive value of 100 per cent and a negative predictive value of 99.5 per cent. The SmartNav system demonstrated high sensitivity and specificity in detecting TFO intra-operatively in patients with normal cochlear anatomy.
- A strong correlation was observed between SmartNav and X-ray imaging in angular insertion depth measurements, supporting its accuracy. Given its advantages such as reduced procedure time and avoidance of intra-operative radiation, SmartNav appears to be a practical and efficient tool in cochlear implantation.
- SmartNav should be regarded as a practical complementary tool in patients with normal cochlear anatomy, while imaging techniques remain the gold standard for detecting TFO.

## Conclusion

The SmartNav system demonstrated high sensitivity and specificity in detecting TFO intra-operatively in patients with normal cochlear anatomy. A strong correlation was observed between SmartNav and X-ray imaging in angular insertion depth measurements, supporting its accuracy. By enabling intra-operative detection and immediate correction of TFO, SmartNav has the potential to reduce the need for revision surgery and the risks associated with it. Given these advantages, SmartNav should be regarded as a practical complementary tool in patients with normal cochlear anatomy, while imaging techniques remain the gold standard for detecting TFO. Further studies are warranted to evaluate its effectiveness in patients with cochlear anomalies and across different implant models.

## Figure 1 Long description

The image shows an X-ray of a cochlear implantation procedure. Key anatomical points are labeled: M for modiolus, RW for round window and S for superior semicircular canal. A green line represents the zero degree reference line between the modiolus and the round window. A dashed line extends from the modiolus, indicating angular insertion depth. The image includes circular markings around the modiolus, highlighting the electrode's position relative to the reference line.

Navigate back to Figure 1.

## Figure 2 Long description

The image A shows an intra-operative X-ray of a cochlear implantation case, highlighting the electrode array. The image B displays a SmartNav interface used during the surgical session. It shows a diagram of the cochlear implant with affected electrodes labeled E19 to E22. The interface includes options like 'Retest Placement Check' and 'Impedance Check'. A graph for time and speed of insertion is present but data is unavailable. The cochlear diameter is noted as 9.2 millimeters.

Navigate back to Figure 2.

## Figure 3 Long description

The image A shows an X-ray of an electrode array positioned within a cochlea. The image B displays a SmartNav diagnostic screen. It includes a diagram of the electrode array with green dots indicating placement points, labeled with angular depth of 324 degrees and cochlear diameter of 9.2 millimeters. The placement check shows '0K' with no affected electrodes. Below, a graph plots time and speed of insertion, with average speed noted as 0.85 millimeters per second.

Navigate back to Figure 3.

## Figure 4 Long description

The image A shows a post-operative X-ray image of TFO, displaying the internal structure and positioning of the cochlear implant. The image B shows a SmartNav interface with insertion diagnostics. It includes a diagram of the cochlear implant with marked angular depth of 312 degrees and cochlear diameter of 9.2 millimeters. The placement check indicates 'OK' with no affected electrodes. Below, a graph shows time and speed of insertion, with seconds on the x-axis and speed on the y-axis, highlighting average insertion speed and specific points of interest.

Navigate back to Figure 4.

## Table 1 Long description

The table details characteristics of 163 study participants, including 213 ears, with a mean age of 17.12 years. Gender distribution was 58.3% female and 41.7% male. All participants underwent cochlear implantation, with 52.6% on the right ear and 47.4% on the left. The CI522 model was used in 96.2% of cases. Tip fold-over was mostly absent, with 97.6% showing no issues on both SmartNav and X-ray. SmartNav diagnostic accuracy was high, with 80% sensitivity and 100% specificity. Placement check and insertion times averaged 148.8 and 52.96 seconds, respectively. Angular insertion depth was slightly greater with X-ray (377.47°) compared to SmartNav (356.84°).

Navigate back to Table 1.
